# Visual Privacy by Context: Proposal and Evaluation of a Level-Based Visualisation Scheme

**DOI:** 10.3390/s150612959

**Published:** 2015-06-04

**Authors:** José Ramón Padilla-López, Alexandros Andre Chaaraoui, Feng Gu, Francisco Flórez-Revuelta

**Affiliations:** 1 Department of Computer Technology, University of Alicante, P.O. Box 99, E-03080 Alicante, Spain; E-Mails: jpadilla@dtic.ua.es (J.R.P.-L.); alexandros@dtic.ua.es (A.A.C.); 2 Faculty of Science, Engineering and Computing, Kingston University, Penrhyn Road, KT1 2EE Kingston upon Thames, UK; E-Mail: F.Gu@kingston.ac.uk

**Keywords:** privacy, context, intelligent monitoring, ambient-assisted living

## Abstract

Privacy in image and video data has become an important subject since cameras are being installed in an increasing number of public and private spaces. Specifically, in assisted living, intelligent monitoring based on computer vision can allow one to provide risk detection and support services that increase people's autonomy at home. In the present work, a level-based visualisation scheme is proposed to provide visual privacy when human intervention is necessary, such as at telerehabilitation and safety assessment applications. Visualisation levels are dynamically selected based on the previously modelled context. In this way, different levels of protection can be provided, maintaining the necessary intelligibility required for the applications. Furthermore, a case study of a living room, where a top-view camera is installed, is presented. Finally, the performed survey-based evaluation indicates the degree of protection provided by the different visualisation models, as well as the personal privacy preferences and valuations of the users.

## 1. Introduction

Assisted living is a very relevant topic at the moment, since more and more older people rely on professional or informal care, and such a service is not always easy to provide at home or in care centres, due to the lack of means. Therefore, it is desirable to enhance and extend people's own autonomy. In the next few decades, a strong demographic change is expected, and a greater number of individuals in long-term care will live alone [1,2]. Nowadays, in order to provide care to this collective, new solutions have to be developed taking into account their needs. Ambient-assisted living (AAL) applications stand out among the possible solutions. AAL aims to support people's health and safety in order to increase their autonomy and well-being. This is achieved by using information and communication technologies (ICT) to provide services, such as automatic supervision of medication or intelligent monitoring [3,4].

Video cameras are being used in AAL more and more frequently. Computer vision techniques allow one to monitor an environment and report on visual information, which is generally the most direct and natural way of describing an event, a person, an object, actions and interactions [5]. These advances have given video cameras the ability of ‘seeing’, thereby becoming smart cameras [6]. They are used for several applications, from tasks, such as face recognition, people identification, object recognition and tracking, to recognition of actions and activities of daily living or even human behaviour analysis during a long period of time [7]. These new abilities allow the development of novel AAL services related to intelligent monitoring for people in need of care. For instance, a service for the detection of home accidents that triggers alarms in order to warn the family or the emergency services. Such services are usually provided, with limited performance, using other types of devices, such as binary state or environmental sensors. Furthermore, by means of the early recognition of behaviour anomalies, signs of cognitive impairment could be detected [8]. Video cameras allow one to obtain a huge amount of environmental data in a non-intrusive and straightforward way. However, the usage of these devices in private spaces brings up ethical concerns related to the privacy of their inhabitants. In that sense, trust and privacy must be central requirements in the design and development of video-based home care solutions [9].

The deployment of smart cameras in private spaces, e.g., family home, nursing home, hospitals, *etc.*, threatens privacy protection and, likely, infringes on people's right to privacy [10]. *A priori*, it is not reasonable to use cameras in private spaces. Surely, inhabitants would feel constantly observed, and in some way, they would be renouncing their privacy right. Indeed, the usage of consumer electronics products like, for instance, cameras in smartphones, wearable cameras and the like or even small drones have raised suspicion. This is due to the abilities of these technologies concerning continuous video recording and people being recorded without consent in both public and private spaces. Therefore, there are some questions that need to be solved before using smart cameras in private spaces: What usage are the cameras for? What information do they get? Could somebody be watching the scene? Who? What could be seen? Is there a visible indicator warning subjects that they are being watched? Could the subject see what is being watched? Could the cameras be disconnected? Are there recordings? How are they used? Has the subject any control? Is the recorded data shared? etc.

Some of the mentioned questions are related to privacy management, whereas others are related to security (access control and confidentiality). This is a very important aspect. A non-authorised intrusion could avoid access control and spy on people without their consent. Equally important is to prevent abuses of the system by operators themselves. These and other questions related to privacy must be taken into account from the early stages of the design of any monitoring system involving people by following the “privacy by design” (PbD) approach [11]. Furthermore, solving these privacy issues related to the use of cameras in private environments will increase the acceptance of novel video-based AAL services [12]. In this paper, a proposal of a level-based visualisation scheme that aims to solve some of these privacy-related questions is presented. Specifically, this paper manages privacy protection in those cases in which someone, for instance from a telecare centre, is accessing the video.

A privacy-by-context approach is introduced that ensures privacy protection by means of the development of several visualisation models that conceal sensitive information and provide different levels of protection. This makes it possible to apply visual monitoring for telecare and telerehabilitation services, among others. Furthermore, a case study of the proposed implementation in a smart lab is detailed. Experimental results based on a survey that has been carried out show that this proposal enables the protection of visual appearance and identity with regard to the subjective perception of the users.

The remainder of this paper is organised as follows: Section 2 details the related work corresponding to visual privacy methods, as well as the background on current needs for advancements. Section 3 presents our proposal of the mentioned scheme as a part of a monitoring system that aims to provide AAL services protecting the individual's privacy. In Section 4, our proposal is implemented as a software prototype containing eight visualisation models. Moreover, a case study based on a smart lab is presented. Section 5 details the evaluation survey that has been carried out regarding its definition and results. The flaws and disadvantages of this approach are discussed in Section 6. Finally, Section 7 presents conclusions and future work.

## 2. Background

The protection of individual's privacy appearing in videos and images is known as visual privacy protection. This topic has been considered in valuable reviews about ambient intelligence (AmI) and AAL, because of its importance for the adoption of video-based AAL applications [8,13]. Visual privacy is also considered in the context of video surveillance applications and other fields [10,14,15]. Video cameras enable the acquisition of a huge quantity of information that can be easily interpreted by human beings in order to extract knowledge. Because of this, people's concerns about privacy arise, and the following question is raised: How can visual privacy be protected? An answer to this question is needed in order to enable the development of novel video-based applications in the near future.

Currently, there is a variety of methods to protect visual privacy in imagery data focusing on privacy-related processing and visualisation issues. Indeed, some methods even try to prevent images from being captured. Because individual's privacy is preserved when there is no matching between person identification and sensitive information, protection methods usually focus on identity protection, for instance masking or blurring faces and number plates. Visual privacy protection methods can be classified into five large categories [16]: (i) intervention; (ii) blind vision; (iii) secure processing; (iv) redaction; and (v) those based on data hiding. In the following paragraphs, each category is described.

Intervention methods aim to create capture-resistant spaces in order to prevent someone from capturing visual data from the environment [17–19]. In order to achieve this, there are two approaches depending on whether there is control enforcement over the capture devices or not. In the first case, the firmware of a camera or an application installed on a smartphone could be responsible for preventing the capture of certain environments. Whereas, in the second case, a specialised device could intervene in the camera optical lenses in order to prevent acquisition.

Regarding blind vision, these methods have to do with image or video processing in an anonymous way, addressing privacy-related processing issues by using secure multi-party computation techniques that are applied to vision algorithms [20,21]. This way, imagery data can be processed using a third-party vision-based algorithm without letting the algorithm know anything about the data, and *vice versa*.

Secure processing is similar to blind vision in the sense that it is used to process information. However, privacy requirements are less constrained. For instance, private content-based image retrieval can be considered as a method of secure processing [22]. In this scenario, a similarity search is carried out in a public database of images where the query image is kept secret. In general, methods that use imagery data in a privacy respectful way are considered under the umbrella of secure processing.

In visual privacy protection, redaction methods stand out as the ones most commonly used. They focus on solving privacy-related visualisation issues. Their principles are simple. They basically modify an existing image to conceal or remove sensitive information in order to preserve privacy. Because individual's privacy is preserved when there is no matching between person identification and sensitive information, redaction methods usually focus on identity protection, for instance masking or blurring faces and number plates. Nevertheless, if image understanding is necessary, there are some restrictions that must be considered regarding how images are modified. This is known as the privacy-intelligibility trade-off. For instance, if a privacy-protected image is needed to assess risks or home accidents in an AAL environment, the modified image has to keep relevant information after being modified in order to be useful. Because of this, redaction methods often need to balance privacy and intelligibility. Besides, there are different techniques that can be used to modify images, for instance image filtering [23], encryption [24], face de-identification [25], object removal [26] and replacement [27] by means of image inpainting techniques and visual abstractions, respectively.

Finally, in order to provide reversibility, *i.e.*, the modification can be undone to recover the original image, data hiding methods are used along with redaction methods [28,29]. Protection methods based on data hiding use techniques like steganography and digital watermarking to conceal an image as embedded data inside another one. The hiding process is controlled by a key or pass phrase that enables the owner of such a key to recover the embedded data. Although reversibility is not required for AAL applications, it may be needed in some scenarios, where original images may be accessed eventually or required as evidence at a trial.

In our case, given that the proposed visualisation scheme focuses on those situations in which access to video is needed by formal caregivers (professionals) or informal ones (family, friend, neighbour, *etc.*) as part of an AAL service, we employ redaction for visual privacy protection. Indeed, we cannot employ intervention, because image acquisition is a requirement in this kind of service. Similarly, blind vision and secure processing methods are not used, because these approaches are related to privacy processing issues and do not solve the ones related to visualisation.

One important aspect of visual privacy protection is the evaluation methodology used to test redaction methods. Due to the subjectivity involved in privacy, *i.e.*, it depends on each individual, its evaluation is not straightforward. There are two approaches for evaluating redaction methods: evaluation based on surveys or based on algorithms. The first approach is based on conducting interviews and questionnaires with users where standard questions are made regarding protected images. Therefore, they evaluate what information can be extracted from these images [30]; whereas, in the second approach, computer vision algorithms are used to obtain a few indicators or metrics from the image in order to evaluate how well the protection works [31]. For instance, the detection and recognition of human faces, bodies, and so on, can be used to obtain recognition rates before and after image modification. Although evaluation based on algorithms provides reproducibility, enabling a fair comparison between redaction methods according to previously established indicators, the detection of such indicators is not always a trivial task. Conversely, evaluation based on surveys enables fine-tuned questions that measure whatever attribute that can be visually perceived by human beings, at the cost of adding subjectivity. Since the indicators needed for the evaluation of our work are very complex to be estimated through computer vision, we have chosen an evaluation based on surveys taking advantage of the flexibility to set the attributes.

## 3. Proposal

Providing a balance between privacy and intelligibility has been briefly discussed in the previous section. There must exist a trade-off between these two in order to protect privacy and, at the same time, preserve information needed for image understanding. However, it turns out that obtaining such a balance is difficult to achieve, if not impossible. In fact, most of the redaction methods reviewed in the state-of-the-art do not successfully solve this issue. For instance, encryption methods provide the highest privacy, but the lowest understanding. When the image is decrypted, this is inverted, obtaining the lowest privacy, but the highest understanding. In the case of image filters, they cannot provide such a balance [32]. Furthermore, this is almost the same situation for the other redaction methods. As it seems that privacy and intelligibility cannot be provided at the same time, we have lowered this restriction in order to propose a practical solution that changes this balance according to the context [33].

Our proposal is an extension of the work presented in [34]. In that paper, we introduced a paradigm for people monitoring that considered privacy from early stages on following a privacy-by-context approach, where privacy requirements were taken into account. In the present work, we have extended the previous one with an extended state-of-the-art, where methods for protecting visual privacy have been reviewed. Furthermore, a use case of a top-view scenario using RGB cameras is presented. This has been complemented with an evaluation of the developed protection methods. In the developed level-based visualisation scheme, privacy is protected by means of a set of redaction-based visualisation models that modify the raw image in order to conceal sensitive information of the subject. The use of a specific model is determined in advance by the user according to the context. In this way, our privacy-by-context approach allows one to adapt privacy to the individual and provides a different balance when needed. We aim to use the proposed scheme in our system [35] as the basis for developing video-based AAL services oriented to care of people, by means of making intelligent visual monitoring and privacy compatible. Furthermore, this work is aligned with the roadmap of the AALIANCE2 project [36], an european alliance that establishes the research priorities in the field of AAL for the next few decades. Concretely, privacy management is one of the issues that must be taken into account in future AAL services for Europe.

### 3.1. Privacy Requirements of the Monitoring System

There are three stages in which privacy protection may be involved. The first one has to do with video transmission through the network; the second one concerns data processing, whereas the third one is related to video visualisation by a viewer. This work is mainly focused on the latter.

The level-based visualisation scheme that is proposed has been mainly designed to work with video-based intelligent monitoring systems for long-term care of elderly or disabled people in private environments, like, for instance, a smart home. Concerning this, and for the sake of clarity, let us explain briefly the requirements of our monitoring system in order to have a better understanding of the problem that the visualisation scheme aims to solve. Our monitoring system is focused on events that put people's health and safety at risk, e.g., falls, home accidents, health problems, and so on. Whenever such an event is detected, the system will trigger an alarm in order to get support for the assisted person. An alarm can be local, in order to warn other inhabitants; or remote, warning other family members, a telecare centre or an emergency service. The automatic accident detection through scene analysis may not always be correct or, despite being correct, the risk assessment may be underestimated. Therefore, human intervention is necessary in order to assess the real risk. This person, *i.e.*, the observer, will have been previously granted the appropriate permissions by the assisted person. Regarding this matter, whenever an emergency is detected, the viewer will receive an alarm and access the video in order to see the scene. It is in this interaction, between the observer and the system, where privacy protection is required, and this is the issue we address in this work.

Furthermore, our system is deployed inside the house and runs AAL services locally. No data are sent from inside outwards, because the event recognition is performed indoors. Due to this, computer vision algorithms access raw data to perform their task. Only filtered data are transmitted outwards for visualisation purposes. Nevertheless, if event recognition should be carried out externally, *i.e.*, in the cloud, a blind vision approach could be used.

### 3.2. Privacy Protection

In contrast to works where privacy is protected by using blurring or pixelating effects to modify an image [23,37], or other works that operate at the same level, but apply a different visual effect [38], this contribution is more similar to [27], where several ways of displaying an object (*i.e.*, visual abstractions) are proposed according to closeness between objects and viewers. Specifically, a level-based visualisation scheme to protect privacy is proposed. Each level establishes the way in which the video images are modified and displayed and, therefore, the provided protection degree. In this scheme, the appropriate level is dynamically selected according to the context, therefore modifying a non-protected image before it is displayed. The correspondence between a given instance of the context and the visualisation level must be performed by the assisted person in advance.

Regarding the context, it has to provide enough information in order to empower people to adapt privacy to their preferences, in such a way that they can decide by whom, how and when they are watched. In order to decide which variables are part of the context, different privacy requirements of an individual have been considered, like the protection of identity, appearance, location and activity. Visual clues involved in the recognition of these context variables have been taken into account. This leads us to propose a context made up of the following variables: (i) the observer; (ii) the identity of the person (to retrieve the privacy profile); (iii) the closeness between the person and observer (e.g., relative, doctor or acquaintance); (iv) appearance (dressed?); (v) location (e.g., kitchen); and (vi) ongoing activity or detected event (e.g., cooking, watching TV, fall). By using these variables, an individual can select the proper visualization level in any situation that can be modelled by the context (see Table 1). This visualisation scheme aims to provide different balances between privacy protection and intelligibility, so it can be adapted to the individual sense of privacy [39].

## 4. Implementation

**Figure 1:**
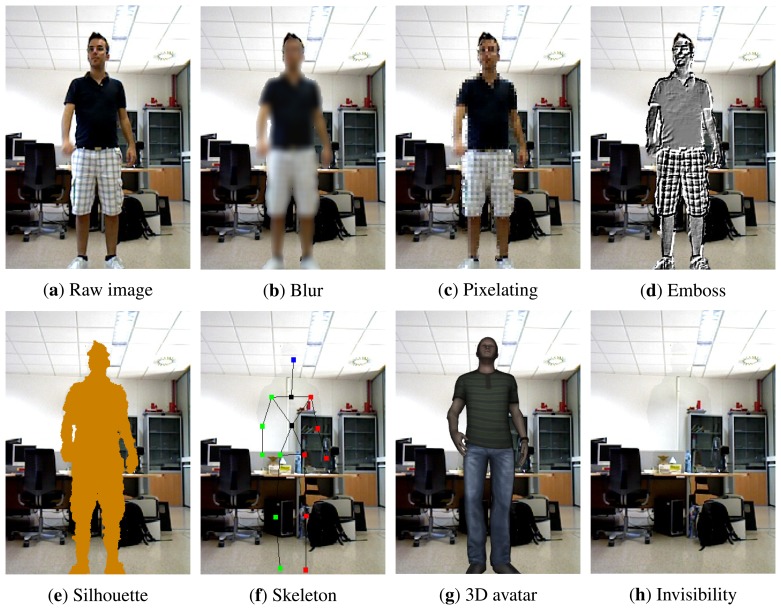
Visualisation models included in our implementation, ordered from lower to higher protection. The results of applying them to the same frame of a video sequence are shown.

In this work, a software prototype for our level-based visualisation scheme has been developed considering eight visualisation models (see Figure 1). Despite these eight models, it is worth mentioning that the proposed visualisation scheme does not define the particular models to be used, but it provides different alternatives that may be considered for each specific application. As can be seen, the silhouette of the person is considered as the sensitive area, thereby these models focus on protecting identity and appearance. The implemented visualisation models use image post-processing methods to modify the image. Specifically, visual effects are applied to the sensitive region to conceal the person, replace the individual with a different representation or even make the subject disappear completely. Next, we describe each visualisation model.

(a) Raw image: This model does not modify the raw image captured by the cameras. Therefore, it does not provide any protection. This visualisation may be necessary in some cases in order to assess the gravity of the detected event.
(b) Blur: A blur effect is applied to the silhouette of the person. This filter does not provide a balance between privacy and awareness [32], but for instance, it is used by Google Street View in order to conceal people's faces and car plates [23].
(c) Pixelating: This model reduces the image resolution. This method is commonly used in TV. Although a balance cannot be provided, admissible results may be obtained by adjusting the level of pixelating [40].
(d) Emboss: This model is suitable to hide colour information of the image (corresponding to skin, hair, *etc.*), but it preserves the structure of the textures. The output is an image where contrast changes in colour are highlighted.
(e) Silhouette: In this case, the model replaces the persons with their silhouette. The information about colour and the structure of textures is removed. Visual clues, like height or the silhouette itself, can still allow the identification of the subject by close people. In this case, nudity is partially protected, since colour is removed, but the shape is preserved.
(f) Skeleton: Using this visualisation model, the subject is replaced with a virtual skeleton that mimics the movements. In this way, the posture of the subject could still be recognised in situations of alarm in order to determine their seriousness. The information about colour and the shape of the person is fully removed, thereby protecting nudity. In this case, just the person's height may allow a direct identification.
(g) 3D avatar: This model replaces the person with a 3D avatar that mimics the posture and movements. Thereby, all kinds of information related to the appearance are removed, preventing direct identification. At the same time, it eases the alarm assessment, because the avatar is a more natural representation than the skeleton. Besides, it is currently possible to create customised 3D avatars by scanning a person [41]. In this way, there may be cases in which the utilisation of a customised 3D model of the person would be acceptable, whereas in others, it would be desirable to use an anonymous 3D model, as in our proposal.
(h) Invisibility: In this last model, the person is completely removed from the image by filling the gap with the background model. In this case, the person could not be directly seen. However, the interaction with the environment could be seen (e.g., objects). Besides protecting the subject, this model could be useful in order to fully conceal non-monitored people appearing in the scene, such as visitors.

Despite the provision of different visualisation models, the privacy preservation of some of them is similar. For this reason, they can be grouped into five visualisation levels: (i) raw image (a); (ii) appearance partially protected (b, c and d); (iii) appearance highly protected (e); (iv) appearance totally protected, *i.e.*, posture only (f and g); and (v) subject is not shown (h).

Regarding technical details, the visualisation models have been developed in C++. The image modification is completely performed on a GPU by using OpenGL ES 2. The model of the background is dynamically updated every frame in order to get a realistic image when the silhouette of the person is removed (Models f, g and h). In this case, we rely on OpenNI2 to access depth information from an RGB-D camera and NiTE2 (this software was publicly available and owned by PrimiSense, Inc. before Apple, Inc. acquired this company) for user tracker and pose estimation in order to recognise the silhouette of the person and the skeleton based on RGB-D data. We have used the PrimeSense 1.09 (short range) device. Using this technology, we have verified that the implemented visualisation models perform above the video frequency (30 fps) without any optimisation. Finally, we are also working in activity recognition to detect the ongoing activity or the event (e.g., home accident) to be used as part of the context [42].

### 4.1. Top-View Scenario

RGB-D cameras provide a horizontal perspective of a monitored environment; their field of views are however limited due the focal length and view angle. In order to grasp the entirety of the environment, one thus would better seek other options, e.g., a wide-angle fish eye RGB camera used to obtain a top-view of the scene. Such a camera, usually installed on the ceiling of a room, for example, is capable of providing up to a 360-degree view angle of the entire room. As a result, this type of camera is ideal for monitoring indoor environments, where localisation of objects or people of interest is the main vision task. Localisation of objects or people using such cameras (*i.e.*, detection and tracking) can be challenging. For instance, for the top-down viewpoint, only the top part (e.g., the head and shoulders) and bottom part (e.g., feet) of a person are visible in most frames, and subjects are often very close to the cameras, so changes of object's appearance can be highly drastic and frequent. Motion-based detection and tracking methods, e.g., background subtraction, can be applied to such scenarios where shapes and appearances of the people and objects of interests are difficult to model. In addition, background subtraction methods are mostly light in terms of computation and, thus, ideal for real-time systems, in which continuous and long-term monitoring is required [43–45].

A single background subtraction method may not be sufficient to deal with situations, such as illumination changes, the subject being static for a considerable length of time, and so on. Therefore, we propose a framework that employs a collection of background subtraction methods that are complementary to each other and that fuses the derived foreground masks at the post-processing stage. The selection criterion is that a background subtraction method should be computational light and robust against environmental changes in the scene, in addition to providing high accuracy in terms of foreground detections. Based on the evaluation in [46] and our own empirical investigation, the Gaussian mixture model (GMM)-based method [47] and multi-cue background subtraction (MCBS) method [48] are selected, by considering the selection criterion above. The GMM-based method employs a motion segmentation algorithm based on an adaptive background subtraction method that models each pixel in the image plane as a mixture of Gaussian models and uses an online approximation to update the model. Each pixel of an upcoming frame is then evaluated against the background to determine its corresponding Gaussian distribution of colour in the background model. Any pixels that do not match the background model are considered foreground, until sufficient and consistent evidence is presented to suggest that a new Gaussian distribution should be created, and the background mixture is then updated. As the identified foreground pixels are rather sparse, a two-pass connected component algorithm [49] is used to segment them into larger regions that correspond to foreground object detections. On the other hand, the MCBS method uses multiple different types of cues, e.g., pixel texture, pixel colour and region appearance. It first clusters the texture information of a given scene via codebook-based background modelling techniques, in order to identify initial foreground regions. The texture features are detected by a texture operator, named the “scene adaptive local binary pattern (SALBP)”, in a consistent and accurate manner. In addition, the colour cue of the background is modelled in the codebook, as well, and used to refine the texture-based detection results through fusing these two types of cues. At last, the appearance of a newly-refined foreground detection is estimated by the partial directed Hausdorff distance between the shape of the foreground boundary and the edge map of the corresponding region in the original input image. Each background subtraction above produces a foreground mask at every frame, which is converted into a bitwise mask (foreground pixels as “1”, while background pixels as “0”) by applying a threshold. Two binary foreground masks are then fused by a simple logic “AND” rule, that is for each pixel in the image plane, only if both binary masks label it as “1”, it is labelled as “1” and “0” otherwise by the fusion process. The resulting fused mask is considered as the output foreground detection of a frame, to which privacy filters can be applied.

**Figure 2:**
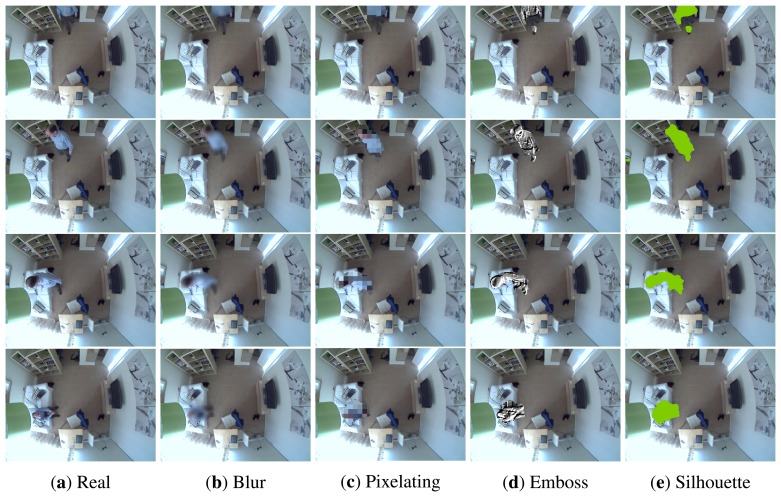
A sequence of images where the real image (**a**) has been protected using a few of the developed visualisation models (**b**–**e**).

We test the proposed framework in an indoor AAL environment, *i.e.*, a living room, where an assisted person is presented in the scene to possibly interact with other objects. A wide-angle fish eye camera is mounted on the ceiling of the room, which provides a 360-degree field of view of its entirety. Due to the privacy requirement in such home spaces, it is ideal for evaluating the proposed privacy filters. By identifying the location of the assisted person in real time, the system can automatically adjust the privacy level for other parties to access the corresponding visual data. In addition, visual activity analysis could provide a visual understanding of the behaviour of the assisted person over time, which can be potentially coupled with an anomaly detection system to raise alarms on suspicious behaviour and potential dangers. Both aspects are essential for improving the quality of the assisted living environment. Figure 2 shows a list of results, where various privacy filters are applied to the detected foreground masks of the assisted person. The framework is developed by incorporating the BGSLibrary [50] and is implemented in OpenCV Version 2.4.10 and gcc Version 4.8.2. All experiments are run on an Intel Core i7-4770, 32 GB RAM PC with Ubuntu 14.04 installed.

## 5. Experimentation

**Figure 3:**
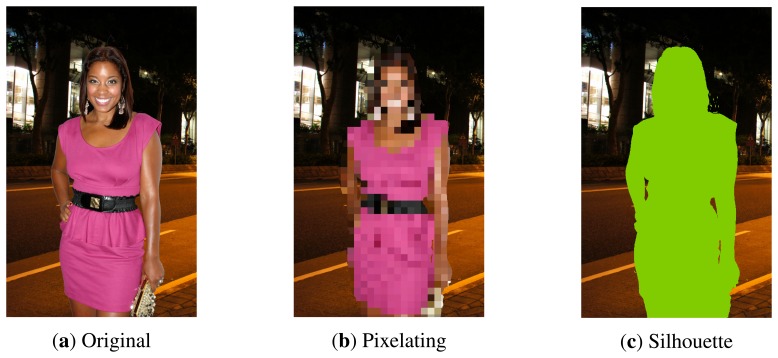
This figure shows three images of the same person that were presented to distinct participants of our survey. As it can be seen, the same image has been modified in different ways leading to two modified images. The original image is licensed under the CC BY 2.0 terms and can be found at http://en.wikipedia.org/wiki/File:Reagan_Gomez-Preston_at_NYTVF.jpg.

For the evaluation of the proposed level-based visualisation scheme, we conducted a survey where 28 individuals participated. A sequence of eight protected images were shown to each participant, and for each image, they were asked to answer several questions about what they were able to see in that image. Figure 3 shows an example of a few images used in the survey. Concerning the used images, we created a dataset with 35 images obtained from Internet services, like Google Images and Flickr, and licensed under the Creative Commons terms. For the creation of this dataset, we browsed these services looking for images containing only a single person with different features, like gender, hair, skin colour, appearance, clothes, and so on, among samples. Furthermore, each image was modified, segmenting the individual and adding a well-known background in order to have separated foreground and background objects. Next, we describe the planning of this survey and present the obtained results, as well as an interpretation of such results.

### 5.1. Planning of the Survey

The survey has been planned according to three main goals: (i) evaluation of the protection provided by the developed visualisation models; (ii) evaluation of privacy preferences regarding the protection of identity, appearance, location and activity; and (iii) evaluation of which visualisation models are preferred by the participants. In order to do so, the survey was divided into two main blocks, one for the evaluation of visualisation models and the other for the evaluation of user preferences. Concerning user preferences, its evaluation was based on two questions where participants were asked to score from one to five the visualisation models, according to which one they thought made it more difficult to see a person, and their privacy preferences, regarding the importance of the protection of identity, appearance, location and activity. However, the evaluation of the developed visualisation models presented a greater challenge.

First of all, we had to establish what was going to be evaluated. Given that the developed methods work over the area bounded by the silhouette of the person, the evaluation of the protection against location inference was discarded. To the same extent, the protection against identification was also discarded. For this purpose, images of persons familiar to the participants where the same person appeared at least once in two different images would have been required, but our dataset did not fulfil this constraint. For this reason, we focused on the evaluation of appearance and activity protection.

After narrowing the scope of the evaluation, the questions that were to be made for the evaluation of both appearance and activity had to be determined. A list of variables related to visual clues of a person's appearance and activity was made, e.g., facial expressions, hair, skin colour, body shape, nudity, clothes, pose, gestures, movement, accessories, and so on. However, only a subset of them was really useful, because, for evaluation purposes, we needed visual items as variables that were measurable. For instance, gender is directly measurable, and it takes values inside the domain [*male*, *female*], whereas pose has a domain whose values are not so commonly well defined. By using these variables, the questions shown in Table 2 were proposed for assessing whether participants could extract the requested information from the images.

Our dataset is composed of 35 images; taking into account that we have eight visualisation models, if all of the them are evaluated with all of the images, this results in 280 modified images and a total of 2520 questions (nine per image). The number of questions had to be reduced to make the survey viable. Several approaches were carried out.

On the one hand, we considered which visualisation models would be worth evaluating using our survey. For obvious reasons, it turns out that the invisibility representation is not well suited for asking question about individual's features. As a result, invisibility is not evaluated in the questions of the first block.

On the other hand, we proposed to use only a subset of the whole image dataset. Testing the visualisation models with only one image would result in the minimum number of questions, *i.e.*, seven modified images that lead to 63 questions. However, if several modified versions of the same image are shown to participants, they can use their memory to recall hidden parts of the image that were visible in other previously presented images. In order to avoid the recall effect completely, the minimum number of different images must be seven, one for each visualisation model. Nevertheless, this way, only a single image would be used for each visualisation model, which does not enable us to make fair comparisons between them. Therefore, we needed to have answers for each image modified by all of the visualisation models. Furthermore, instead of the seven images, we finally used eight images in order to have a well-balanced number of features, e.g., the same number of men and women, the same number of people with similar skin colour, and so on. This way, the resulting bias is avoided.

After considering all of these constraints, we ended up with eight images that were modified using all of the visualisation models, except invisibility. That resulted in 56 modified images leading to a total of 504 questions. However, all of the images could not be presented to a participant to avoid the recall effect, as mentioned before. Therefore, the modified images were split into several partitions where images were not repeated inside a given partition, so that there was no recall effect, and each image was modified differently between partitions in order to use all of the visualisation models. In Table 3, it can be seen how images have been finally organised into seven partitions that fulfil all of the constraints. As can be observed, the real visualisation model is included, because we will use the answers to unmodified images as the ground truth. Furthermore, given that we use eight images instead of seven, one of the visualisation models is repeated in each partition.

Finally, a participant of the survey will have to answer only those questions related to images of one of the seven partitions, *i.e.*, only 72 questions will be presented to each participant. Because of this, the minimum number of participants required to have an answer to each of the questions matches the number of partitions, *i.e.*, seven participants in this case. Nevertheless, more than one answer per question is needed in order to extract conclusions. In this case, the minimum number of required participants has been established as 28 in order to obtain four answers per question.

#### Establishment of Ground Truth

As has been mentioned in the last section, the ground truth employed to evaluate the answers of the participants is based on their own replies to Questions 1–9 when observing the real image without modifications. In this way, our own bias towards assigning specific values can be avoided.

For single choice question, the chosen ground truth value is based on all of the different answers that the participants have submitted, excluding the “I don't know” option. This means that, for instance, if some people indicated a brown hair colour and others a black hair colour, both are considered as ground truth. Regarding the multiple choice questions, each member is considered individually, leading to multiple binary questions. The majority vote of each member is taken as ground truth, unless a draw is detected, in which case, it is assumed that the answer is not clear enough, and it should be ignored for evaluation. In other words, if the majority of the people were able to see, for instance, one of the accessories, it is considered to be present. However, if a draw is generated, we assume that this attribute should not be considered.

The comparison between answers and ground truth is straightforward for the single choice options, *i.e.*, the answer has to match the ground truth. However, for the multiple choice options, we have to take into account that only true ground truth values should be compared to the answers, and false ones should be ignored. For instance, if a person does not wear sunglasses, not detecting them with a specific visualisation model does not lead to a correct answer.

### 5.2. Results

**Figure 4:**
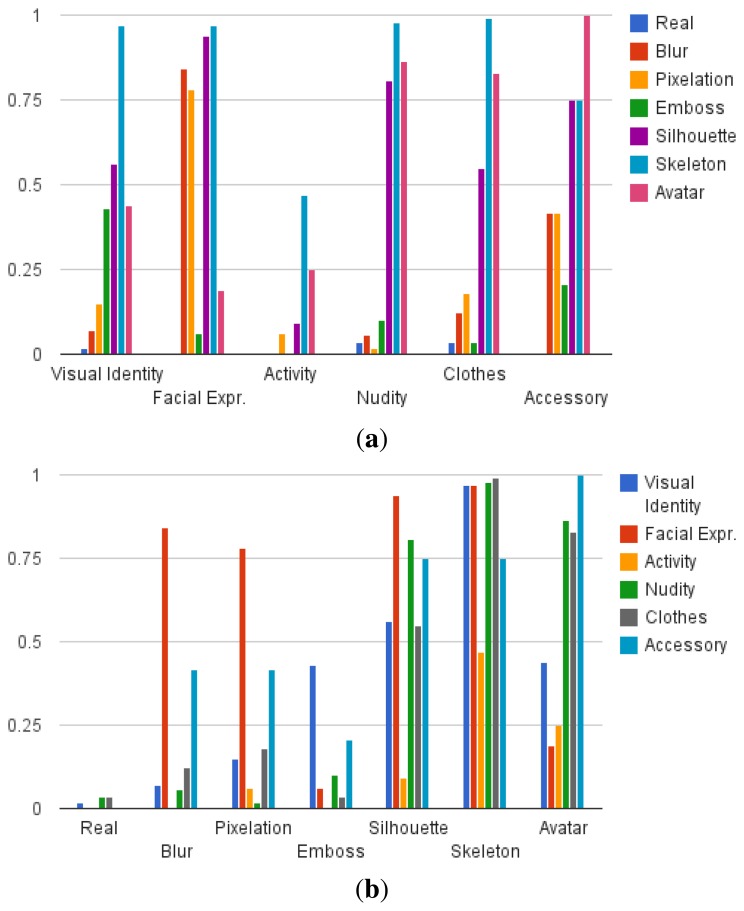
The charts show the average protection degree of the visualisation models for several visual attributes. For an easier comparison, (**a**) shows the information grouped by visual attributes, whereas (**b**) shows the same information grouped by visualisation models (zero equals no protection; one equals full protection).

**Figure 5:**
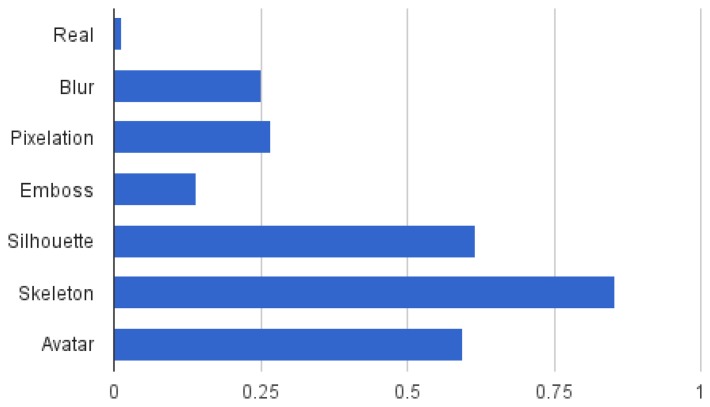
Overall average protection degree of the visualisation models considering all of the attributes.

**Figure 6:**
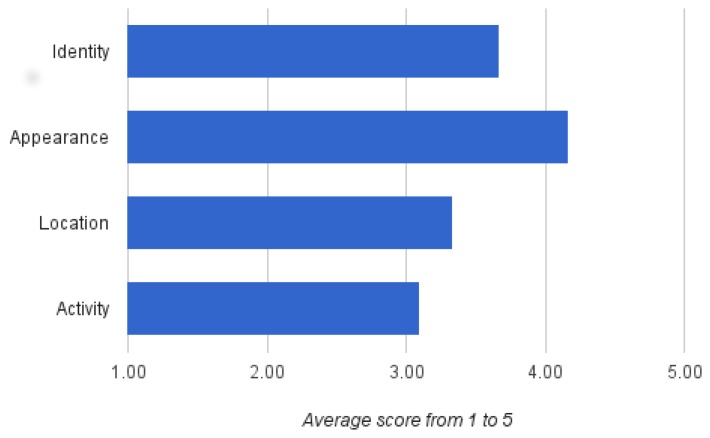
Participants' valuation regarding visual privacy requirements. For each item, participants gave a score between one (not important) and five (very important).

**Figure 7:**
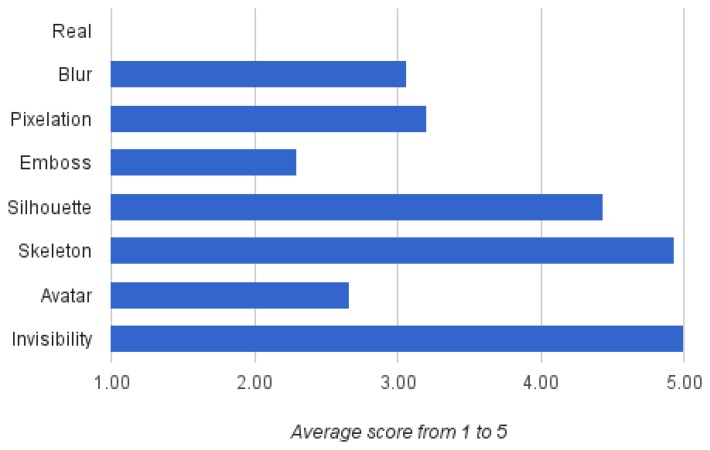
Participant's valuation of the visualisation models regarding the difficulty of seeing the person properly. For each visualisation model, participants gave a score between one (very easy) and five (very difficult).

To ease the interpretation of results, these are presented in charts in Figures 4, 5, 6 and 7. The results of the first part of the survey, where the visualisation models are evaluated, are shown in Figure 4. Once the user answers have been compared to the ground truth, the resulting data show the number of correct and wrong answers for each question, as well as those that should be ignored when averaging, as previously detailed. The average amount of correct answers of a question is interpreted as the detection rate *d_a_* of the specific visual attribute *a* that is evaluated. The resulting protection degree can then be modelled as *p_a_* = 1 − *d_a_*, leading to a value that indicates how well a visual attribute is protected when applying a visualisation model.

Figure 4 shows the average protection degree of the seven evaluated visualisation models grouping the 32 evaluated visual attributes into six different classes (as shown in Table 2). The overall average protection degree is shown in Figure 5. It can be observed that some visual attributes are more difficult to protect in general, such as visual identity, activity and nudity, and that the performance of a visualisation model is not stable across different types of visual attributes. For instance, blur or pixelation achieve significantly better results on facial expression than on other attributes, and avatar stands out for accessories. It is worth mentioning that this is the desired result of the proposed level-based visualisation scheme, since the proposed visualisation models allow one to set different levels of protection.

In general, however, it can be observed that silhouette, skeleton and avatar tend to obtain the best protection, presenting nonetheless a sensitivity to activity. Furthermore, the avatar obtains poor results on visual identity and facial expression compared to the silhouette and the skeleton, which leads to the thought that users have evaluated the avatar itself instead of the substituted person. The poorest results are obtained by the emboss visualisation model, although it outperforms blur and pixelation in protecting the visual identity, which includes attributes, such as skin and hair colour. This is due to the colour loss of this method, which only preserves textures. It can also be clearly seen that depending on the attribute that has to be protected, it might not be necessary to employ a sophisticated visualisation model, and more simple ones, such as blur or pixelation can achieve good results. Overall, the skeleton is the visualisation model that achieves the highest protection based on the user's criteria.

With respect to the second part of the survey, Figure 6 shows the relevance the participants assigned to the protection of their identity, appearance, location and activity on a scale from one to five. Interestingly, only small differences can be detected, and the standard deviation between answers shows that only limited conclusions can be drawn (for the four categories *σ* ∈ [1.14,1.44]). On average, however, appearance attributes, such as nudity and clothes, stand out.

Regarding the visualisation models, users were asked to score how difficult it is for them to see a person correctly using the same image, in this case with all of the visualisation models. Figure 7 details that the invisibility obtains the highest score as expected, but silhouette and skeleton also reach very high scores. Surprisingly, the avatar is below blur and pixelation, even though formally, the same information is shown as in the skeleton. Again, the human appearance of the avatar may have mislead the user's judgement about what can be seen and what is protected with this visualisation model.

In conclusion, from the subjective perception of the participants in this survey, the skeleton visualisation model results as a clear winner in terms of higher protection, presenting both the highest average protection degree, as well as a greater difficulty of seeing the person properly. Nonetheless, privacy protection can also be satisfied using the silhouette or avatar visualisation models, which probably could prove to be more practical for AAL applications. Furthermore, the results of this study also make it possible to choose visualisation models based on specific application needs in order to achieve the sought-after balance between privacy and intelligibility.

Finally, personal details about the users, such as gender, age range and familiarity and usage of the Internet and smart phones, have been collected. Nonetheless, it has been decided to avoid a division of the results by these attributes, due to the lower number of resulting participants.

## 6. Discussion

The visualisation scheme that has been presented relies on the detection of the silhouette of the person. The correct detection of this region of interest is a key aspect for privacy protection. For instance, when processing a sequence of images, if the detection fails in only one single frame, then the protection of the whole sequence will fail, *i.e.*, the person may be identified and tracked in images where privacy was presumably preserved. In our implementation (Section 4) of the visualisation scheme, the silhouette was easily obtained by NiTE2 user tracker software using an RGB-D camera. However, this task has proven to be more difficult using an RGB camera, like the one used in the top-view scenario. In this case, due to the missing depth information, other techniques had to be employed that are not as accurate. Moreover, avatar and skeleton visualisation models are not available when using RGB-only cameras, because of the same reason.

Furthermore, not only the proper detection of the silhouette is important, but also the identification of the person. Although it seems contradictory to have persons identified, it is needed in order to retrieve the privacy preferences of each person. It is worth mentioning that the identity should be used only for that purpose, and human beings should not have access to this information. Nonetheless, we think that identity is difficult to protect in some scenarios, because it may be deduced. For instance, using silhouettes in a typical scenario of a house with three inhabitants, identification using visual clues, like shape, height, and so on, would be possible. Even when the skeleton is used, there is still a 1/3 chance to correctly identify a person. Therefore, we think that in this kind of scenario, the focus should be on appearance protection.

Concerning the evaluation, although the avatar visualisation model has obtained an average protection degree that places it in third position, it was expected to obtain a better score, because formally, the same protection is performed as for the skeleton. As has been mentioned, it seems that users did not take into account that the avatar was concealing an actual person and answered about the avatar itself. For instance, in the raw answers, we have observed that the gender was guessed correctly in 50% of the cases, *i.e.*, only those cases where the actual person was male (as the avatar). This also happened for other attributes.

Regarding the skeleton, it was expected to obtain the maximum score in all of the visual attributes, but activity, because formally, it is not possible to see anything else other than the pose of the person. However, it seems that the backgrounds of the images have provided some context in order to enable participants to perform inferences. In this sense, it is interesting to see how at least two participants have guessed correctly some attributes. For example, one participant guessed that the person appearing in the image was carrying a device, just because of the pose of the skeleton. Another one guessed, based on the background, which showed a running track, that a person was dressed with short trousers and a t-shirt and, consequently, had shoulders and legs uncovered.

It is also worth mentioning that we have learned several lessons during the evaluation. If we had to repeat the survey, we would increase the number of participants to far more than 28 persons in order to have more statistically-significant results. Moreover, it would have been desirable to have more than four answers for each protected image and, preferably, an odd number of answers in order to avoid draws when establishing the ground truth. Regarding the multiple choice questions, we would convert them to single choice questions having three possible answers: “yes”, “no” and “I don't know”. In this way, ambiguity is avoided, and we would be able to evaluate not only the presence of nudity, clothes and accessories, but also their absence.

Nevertheless, after developing and evaluating the level-based visualisation scheme, we firmly consider that this work is a first step towards privacy protection in video that enables a certain degree of understanding about what is happening in the scene. As has been seen throughout this work, this is an important requirement for vision-based AAL services that may require human access to video.

## 7. Conclusions

In this paper, we have presented a privacy scheme that uses visualisation levels in order to display the real image in different ways for the purpose of preserving privacy. The selection of the appropriate level is handled by the assisted person according to the context made up of seven variables. By using this, the individual can decide how he or she is visualised in any situation. As part of our proposal, we have developed a prototype that has eight visualisation models. These are focused on the protection of the identity and the appearance of the person, and they can work in real time. This approach has been deployed in a smart lab scenario where a top-view camera is used for visual monitoring of activities of daily living. With the survey carried out, we have been able to validate that the proposed visualisation models protect people's visual appearance and identity to their satisfaction. This leads to the conclusion that this approach can be applied in homes and care centres, enabling privacy-preserving visual monitoring.

As future work, it would be interesting to compare the different visualisation models, as well as to develop new ones that make use of 3D reconstruction techniques to virtually represent the real scene. Furthermore, other image regions should be considered as sensitive areas (e.g., background and foreground objects), so as to prevent indirect identification through inference channels and previous knowledge [31]. Further research will be carried out in order to recognise identity (through face or gait recognition) and appearance to enhance the context. Moreover, it would be interesting to reduce possible context instances by gathering similar events and activities by level of alarm, as suggested in [51], and observers according to role and closeness to the assisted person, as suggested in Table 1, so as to ease the initial setup of the monitoring system.

## Figures and Tables

**Table 1:** An example of the privacy levels (see Section 4) selected by John according to the context.

**#**	**Observer**	**Rest of the Context**	**Visualisation Level**
1	My daughter Mary (caregiver, relative)	dressed, living room, watching TV, no event	Raw image
2	My daughter Mary (caregiver, relative)	undressed, bathroom, shower, fall	Highly protected (Silhouette)
3	Alice (my doctor, friend)	dressed, living room, watching TV, no event	No image
…	…	…	…
n	Bob (Mary's husband, relative)	dressed, kitchen, cooking, faint	Totally protected (3D avatar)

**Table 2:** This table shows the questions and possible answers of the first block of the survey. A total of 32 visual attributes are evaluated.

**#**	**Class**	**Question**	**Possible Answers (Single Choice Unless Otherwise Indicated)**
1	Visual identity	Could you indicate if the person appearing in the image is male or female?	Male/female/I don't know
2	Visual identity	What is the colour of his or her hair?	Black/brown/blonde/red/white/I don't know
3	Visual identity	Indicate the hair style.	Short/long/I don't know
4	Visual identity	Indicate the skin colour.	White/brown/black/I don't know
5	Nudity	Mark below which parts of the body are nude in the image.	Shoulder/arm/chest/chest (but no breasts)/abdomen/thigh (above the knee)/leg (below the knee) (multiple choice)
6	Facial expression	Could you indicate if the person in the image looks serious or smiling?	Serious/smiling/I don't know
7	Clothes	Mark below which clothes are worn by the person in the image.	Short trousers/long trousers/T-shirt/shirt/jacket/dress/bikini/tie/cap/hat/belt (multiple choice)
8	Accessory	Mark below which accessories are present in the image.	Guitar/camera/sun glasses/folder/bag/bottle/headset/electronic device (multiple choice)
9	Activity	Which of the following activities better match the image?	Standing/walking/running/I don't know

**Table 3:** This table shows how the eight images have been organised into seven partitions (columns). According to the partition, each image is modified using one of the visualisation models.

**Model**	**Part 1**	**Part 2**	**Part 3**	**Part 4**	**Part 5**	**Part 6**	**Part 7**
Real	Image 1	Image 7	Image 6	Image 5	Image 4	Image 3	Image 2
Blur	Image 2	Image 1	Image 7	Image 6	Image 5	Image 4	Image 3
Pixelation	Image 3	Image 2	Image 1	Image 7	Image 6	Image 5	Image 4
Emboss	Image 4	Image 3	Image 2	Image 1	Image 7	Image 6	Image 5
Silhouette	Image 5	Image 4	Image 3	Image 2	Image 1	Image 7	Image 6
Skeleton	Image 6	Image 5	Image 4	Image 3	Image 2	Image 1	Image 7
Avatar	Image 7	Image 6	Image 5	Image 4	Image 3	Image 2	Image 1

**Image**	Image 8	Image 8	Image 8	Image 8	Image 8	Image 8	Image 8
**Model**	Real	Blur	Pixelation	Emboss	Silhouette	Skeleton	Avatar
